# Ten Minutes of Passive Hip and Knee Joint Movement Reduces Stiffness Due to Osgood‐Schlatter Disease in the Rectus Femoris Muscle

**DOI:** 10.1002/ars2.70041

**Published:** 2026-05-21

**Authors:** Takayuki Inami, Naoki Ikeda, Shota Yamaguchi, Genko Karasawa

**Affiliations:** ^1^ Institute of Physical Education Keio University Yokohama Japan; ^2^ Karasawa Orthopedic Clinic Yokohama Japan

## Abstract

**Purpose:**

To investigate the acute effects of passive movement on rectus femoris (RF) stiffness, flexibility, and subjective knee joint pain in patients with Osgood‐Schlatter disease (OSD).

**Methods:**

Patients with bilateral OSD at the orthopedic clinic between July 2023 and June 2025 were prospectively enrolled. One foot was randomly assigned to the intervention group (10 minutes of passive hip and knee joint movement), and the other was assigned to the control group (10 minutes of rest). The acute effects before and after the intervention (PRE and POST, respectively) were compared. RF stiffness was assessed PRE and POST using shear modulus values, heel‐buttock distance, and subjective pain. A stratified cluster analysis of the treatment effect was performed based on the PRE shear modulus values in the intervention group (high‐value and low‐value groups).

**Results:**

Fifty‐three participants diagnosed with bilateral OSD participated in the study. The RF shear modulus significantly decreased following the intervention (21.0 ± 4.4‐17.5 ± 3.2 kPa) (*P* < .001), with the high‐value group (–20.1 ± 9.6%) showing a significantly greater effect than the low‐value group (–13.8 ± 8.6%) (*P* = .022). Depending on the threshold, 83.0% exceeded the minimal detectable change at the 95% confidence level, and 86.8% exceeded the smallest worthwhile change (0.2 standard deviation), while 66.0% exceeded the more conservative smallest worthwhile change (0.5 standard deviation). Heel‐buttock distance and subjective pain significantly improved after passive movement (heel‐buttock distance: 12.5 ± 4.7 to 9.3 ± 3.4 cm; subjective pain: 4.2 ± 2.4 to 2.6 ± 2.0 cm) (both *P* < .001). None of the parameters changed significantly in the control group.

**Conclusions:**

Ten minutes of passive hip and knee joint movement reduced RF stiffness in patients with OSD and improved knee joint flexibility and subjective pain. Furthermore, passive movement showed the greatest effect in the high‐value shear modulus group.

**Level of Evidence:**

Level II, prospective comparative study.

## Graphical abstract

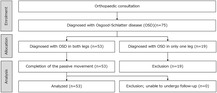

Osgood‐Schlatter disease (OSD) is common in active young individuals and is characterized by traction apophysitis of the tibial tubercle.^1^, ^2^ A systematic review^2^ reported that muscular factors play a clear role in the etiopathogenesis of OSD. In particular, increased tightness of the quadriceps femoris muscle has been consistently reported as an associated risk factor for OSD.^3^, ^4^, ^5^, ^6^ Given that OSD is associated with sports that involve repetitive strain on the patellar tendon and tibial tubercle, including jumping, squatting, kicking, and running,^7^, ^8^, ^9^ it is logical to focus on the properties of the rectus femoris (RF) muscle, such as muscle stiffness. Muscle stiffness has been defined as the ratio of change in force to change in length along the muscle's longitudinal axis, and it is linearly correlated with contraction intensity.^10^ Maintained muscle stiffness leads to excessive contractile activity and increased tightness.

Changes in muscle stiffness have been evaluated using joint angle‐torque relationship and range of motion tests, including assessing tightness (e.g., heel‐buttock distance [HBD]); however, these measurements represent a composite resistance of both muscle and nonmuscle tissues, such as tendons, ligaments, and skin, and do not directly reflect muscle stiffness.^11^ Consequently, the use of ultrasonic shear‐wave elastography (SWE) to directly measure muscle stiffness has increased. The in vivo muscle shear modulus measured by SWE is used to noninvasively analyze the stiffness distribution of muscle tissue,^12^ which can be safely used for repeated measurements.^11^ However, to our knowledge, only 1 study has investigated the relationship between quadriceps muscle stiffness and OSD assessed using SWE. Enomoto et al.^13^ compared the stiffness of the RF during passive knee flexion using SWE in 28 legs affected by OSD and 26 without OSD. The results showed that (1) RF stiffness (shear modulus calculated from the shear‐wave velocity, assessed by ultrasonic SWE) in children with OSD was higher than in children without OSD and (2) children with OSD had increased RF muscle stiffness under stretched conditions (knee joint at a 45° and 90° of flexion) compared with the nonstretching condition (knee joint at 0° extension). This implies that the underlying RF stiffness in patients with OSD contributes significantly to the stress associated with RF stiffness. Although knowledge of RF is limited, an exponential increase in the shear modulus during passive stretching has been reported for other muscles, including the gastrocnemius and soleus.^14^, ^15^, ^16^, ^17^, ^18^

According to a recent review of OSD, conservative treatment options include thorough joint mobilization and stretching.^1^, ^2^ Joint mobilization is defined as a low‐velocity/high‐amplitude intervention.^19^ According to a systematic review and meta‐analysis, joint mobilization techniques may have a pain‐modulating effect by eliciting hypoalgesic responses, as evidenced by decreased pain.^20^ In contrast, various methods are used for stretching, avoiding increases in active traction stress from the RF and RF stiffness characteristics; stretching involving large joint movements, such as the most representative hip extension and maximum knee flexion, may not be recommended. Interestingly, passive movement, which broadly encompasses joint mobilization and stretching, can lead to increased passive stretch.^21^ Muscle overload caused by passive movement is a relatively small stimulus. However, it has been shown to enhance muscle blood flow without a concomitant increase in muscle metabolism.^21^ Although passive movement may be useful for improving RF stiffness in patients with OSD, its effect on stiff RF in these patients remains unclear.

The purpose of this study was to investigate the effects of passive movement on RF stiffness, flexibility, and subjective knee joint pain in patients with OSD. We hypothesized that passive movement would reduce RF stiffness and improve scores on the HBD test and subjective pain in patients with OSD and that these effects would vary among individuals, given the relatively small mechanical load of passive movement.

## METHODS

### Experimental Design and Participants

**FIGURE 1:**
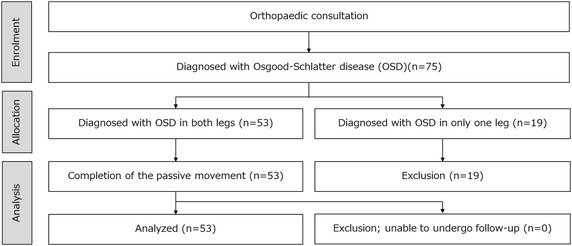
Flowchart illustrating study enrolment. (OSD, Osgood‐Schlatter disease.)

This study was conducted in accordance with the principles of the Declaration of Helsinki and was approved by the university ethics committee. Prior to participation, the purpose, content, method, and risks of the study were explained to the children and their guardians, and informed consent was obtained. Patients seen at the orthopedic clinic between July 2023 and June 2025 who had ultrasound findings of bilateral OSD, based on a previous study, were evaluated for inclusion^22^, ^23^ (Figure 1). Exclusion criteria were patients who were diagnosed with OSD in only 1 leg and those who were unable to undergo follow‐up after passive movement. An orthopaedic surgeon (G.K.) assessed pain during exercise, a protuberance, and tenderness at the tibial tuberosity. OSD was defined as the presence of one or more of these factors, along with ultrasonographic evidence of free bone fragments on the tibial tuberosity and cartilage swelling. Free bone fragments were defined as hyperechoic structures observed within otherwise hypoechoic cartilage (excluding secondary ossification centers), and cartilage swelling was defined as an irregular surface on the skin caused by bulging cartilage. Ultrasound examinations were performed with the knees flexed to 90° using the Sonimage MX1 system (Konica Minolta, Japan) and an HFL38/13‐6 probe positioned at the tibial tuberosity, capturing long‐axis views of the tibial tuberosity. One foot was randomly assigned to the intervention group (10 minutes of passive hip and knee joint movement), and the other was assigned to the control group (10 minutes of rest). The acute effects of passive movement were then compared before and after the intervention (PRE and POST, respectively). A stratified cluster analysis of the treatment effect was also performed based on the PRE shear modulus values in the intervention condition to verify the detailed magnitude of the intervention effect.

### Passive Movement Intervention

**FIGURE 2:**
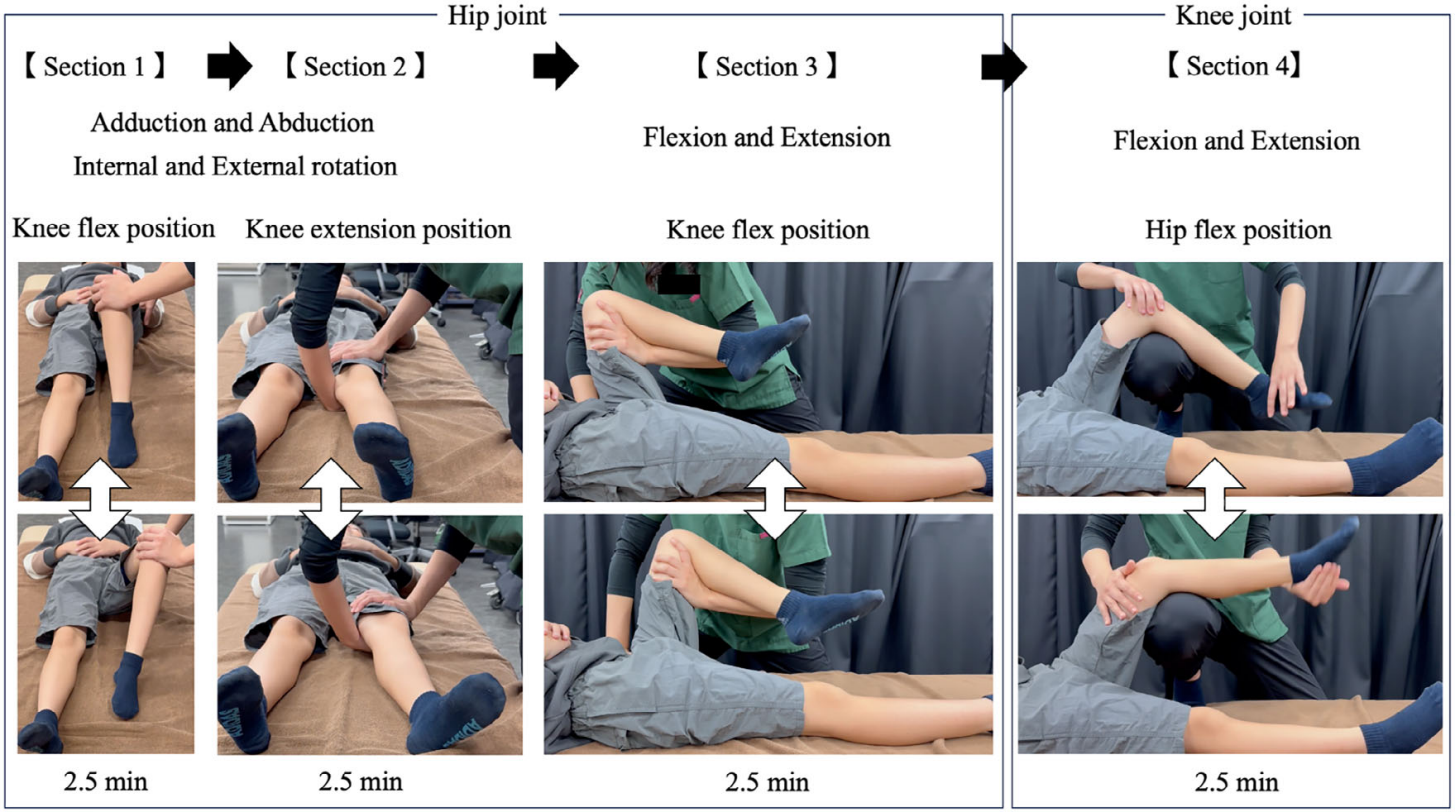
A detailed description of the passive movement method used in this study.

Passive movement was conducted in a temperature‐controlled room. Participants were placed in the supine position on the treatment plinth. As previously described,^24^, ^25^ participants underwent an initial 10‐minute resting period. Subsequently, a 10‐minute intervention was set for the legs in the passive condition. Passive movement intervention was applied to the hip and knee joints based on RF anatomy, modified based on the methods of Hellsten et al.^21^ As shown in Figure 2, passive movement was performed in 4 stages. First, the subject passively moved the hip joint from a 90° knee flexion position through approximately 0° to 45° of adduction and abduction. The second section involved moving the hip joint from a 90° knee flexion position through approximately 0° to 45° of internal and external rotation. This was repeated. Third, the subject passively extended the hip joint from a 90° flexed position to approximately 0° to 30 or 45°, paying attention to anterior pelvic tilt. This was repeated. In the fourth and final section, the subject passively extended the knee joint from a 90° flexed position to 45° of flexion. A metronome set to 50 to 60 cycles per minute was used to control the rhythm of each section. Each section was separated by 2.5 minutes, for a total of 10 minutes of passive movement. The same expert therapist (≥5 years of experience) performed repetitions of these passive movements. In the control condition, participants were instructed to rest in a supine position on the bed for 10 minutes, without moving their legs.

### Shear Modulus of the RF Muscle

**FIGURE 3:**
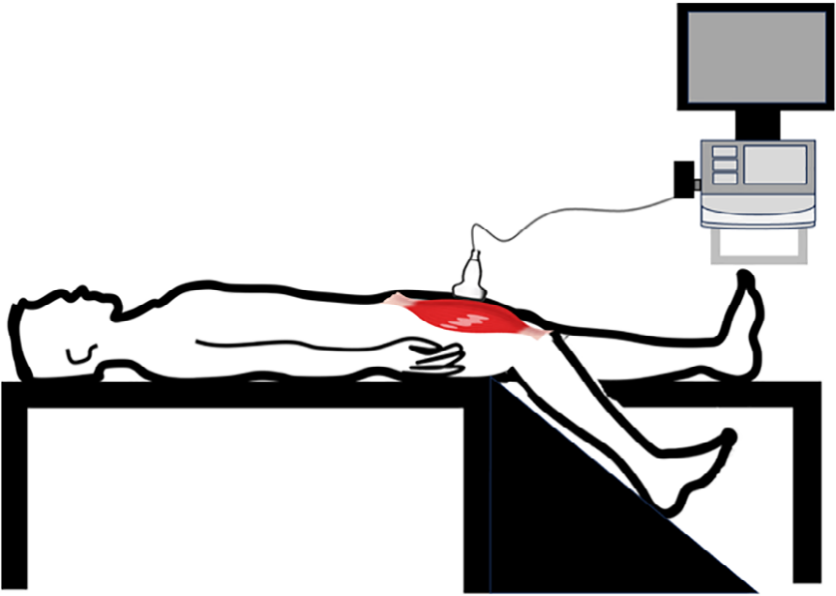
Experimental setting. The shear modulus of the RF was measured at 45° of knee joint flexion (defined as full extension: 0°) while lying supine on a custom bed, using the ultrasound system in shear‐wave mode with a linear transducer. (RF, rectus femoris.)

Based on a previous study,^13^ the shear modulus of the RF was measured at 45° of knee joint flexion (full extension, 0°) while lying supine on a custom bed (Figure 3), using the Aplio 300 ultrasound system (Canon, Japan) in shear‐wave mode with a linear transducer. The probe was placed over the thigh muscle belly, approximately halfway between the greater trochanter and the knee joint. Using a semipermanent ink marker, the probe was positioned at the same location for the PRE and POST measurements. Throughout the scan, care was taken not to press or deform the muscles. The room temperature was maintained at 25°C throughout the measurements. Measurements of the RF shear modulus were performed within 1 minute of the passive movement intervention.

A rectangular region of interest was defined for each long‐axis B‐mode ultrasound image, and the mean shear modulus within the region of interest was recorded as the data. The mean shear modulus was calculated using built‐in software. RF stiffness was defined as the mean of 3 shear modulus values obtained from the 3 ultrasound images. All measurements and analyses of the ultrasonography data were performed by an experienced examiner (>20 years of experience).

### HBD and Subjective Pain

Based on previous studies,^3^, ^4^, ^5^, ^26^ the HBD, as a test for quadriceps muscle tightness, was measured in the prone position on the bed with the legs aligned. The examiner held the patient's ankle and passively flexed the knee joint from full extension (180°), 1 leg at a time. The HBD was measured using a tape measure when the patient reported knee pain or discomfort. The coefficient of variation of HBD was 1.4% in 10 measurements on the same participant (n = 8).

To evaluate subjective pain, we measured the tibial tuberosity pain during knee joint flexion and extension (slow squats using body weight) with the hands placed on the wall. Subjective pain was assessed using a 100‐mm visual analog scale (VAS), with 0 indicating no pain (or no fatigue) and 100 indicating extreme pain (or extreme fatigue). Patients were instructed to interpret a score of 100 as representing the most intense traction pain they had ever experienced at the tibial tuberosity due to OSD. Subjective pain was measured when the patients returned (extended) their knee joints. The coefficient of variation of subjective pain was 1.9% in 10 measurements on the same participant (n = 8).

### Statistical Analysis

All data are expressed as mean ± standard deviation (SD). Correlations between variables were analyzed using Pearson's product‐moment correlation. Raw data were used for the correlation analysis of subjective pain. For other measurements, percentage changes were used for the correlation analysis. Changes after intervention were compared between conditions (passive movement vs control condition) using a 2‐way analysis of variance with 2 factors (condition × time). Additionally, group comparisons (high vs low) were conducted using a 2‐way analysis of variance with 2 factors (group × time) to assess PRE shear modulus values and the intervention effect. If a significant interaction effect was found, a post hoc test was performed to identify the time points with significant differences between conditions using Bonferroni's method. Statistical significance was set at *P* < .05.

A stratified cluster analysis divided participants into groups with high and low PRE shear modulus values in the intervention condition to assess the magnitude of the intervention effect. All statistical analyses were performed using Predictive Analytics Software version 28 for Windows (SPSS Japan, Tokyo, Japan). A priori statistical power analyses indicated that this study design would require 14 participants per condition (repeated‐measures analysis of variance within factors; effect size, 0.4; power, 0.8; alpha level, 0.05),^27^ using G*Power 3.

To interpret the clinical relevance of changes in the RF shear modulus, we used a distribution‐ and error‐based approach because no anchor‐based thresholds or prior minimal clinically important difference (MCID)/patient acceptable symptom state/substantial clinical benefit values were available for OSD. Test‐retest reliability (intraclass correlation coefficient^1^, ^2^) was estimated in a subset under identical conditions. The standard error of measurement and the minimal detectable change at the 95% confidence level (MDC_95_) were calculated. We further defined the smallest worthwhile change (SWC) a priori as 0.2 and 0.5 of the baseline SD and conducted sensitivity analyses across these thresholds. When concurrent clinical outcomes were available (e.g., pain VAS), we derived an indirect (exploratory) MCID for stiffness by regressing delta VAS on delta stiffness and mapping literature‐based VAS MCID values onto the model predictions.

## RESULTS

A total of 53 patients (47 males and 6 females, with a follow‐up of 1 day) were included in this study after excluding those without follow‐up data and those diagnosed with OSD in only 1 leg (n = 19; Table 1). All patients participated in sports activities (baseball [n = 16], soccer [n = 9], basketball [n = 9], rugby [n = 3], track and field [n = 3], badminton [n = 2], tennis [n = 1], dance [n = 1], gymnastics [n = 1], kendo [n = 1], American football [n = 1], competitive karate [n = 1], handball [n = 1], dodgeball [n = 1], karate [n = 1], softball [n = 1], and volleyball [n = 1]) at mean of 2 to 3 times per week.

**TABLE 1:** Patient Demographics in This Study

	Age	Height	Weight
47 males	12.4 ± 2.0 yr	155.0 ± 13.4 cm	47.2 ± 12.9 kg
6 females	11.7 ± 1.6 yr	147.8 ± 8.6 cm	37.3 ± 8.8 kg

*Note*: Values are expressed as mean ± standard deviation.

cm, centimeter; kg, kilogram; yr, year.

**FIGURE 4:**
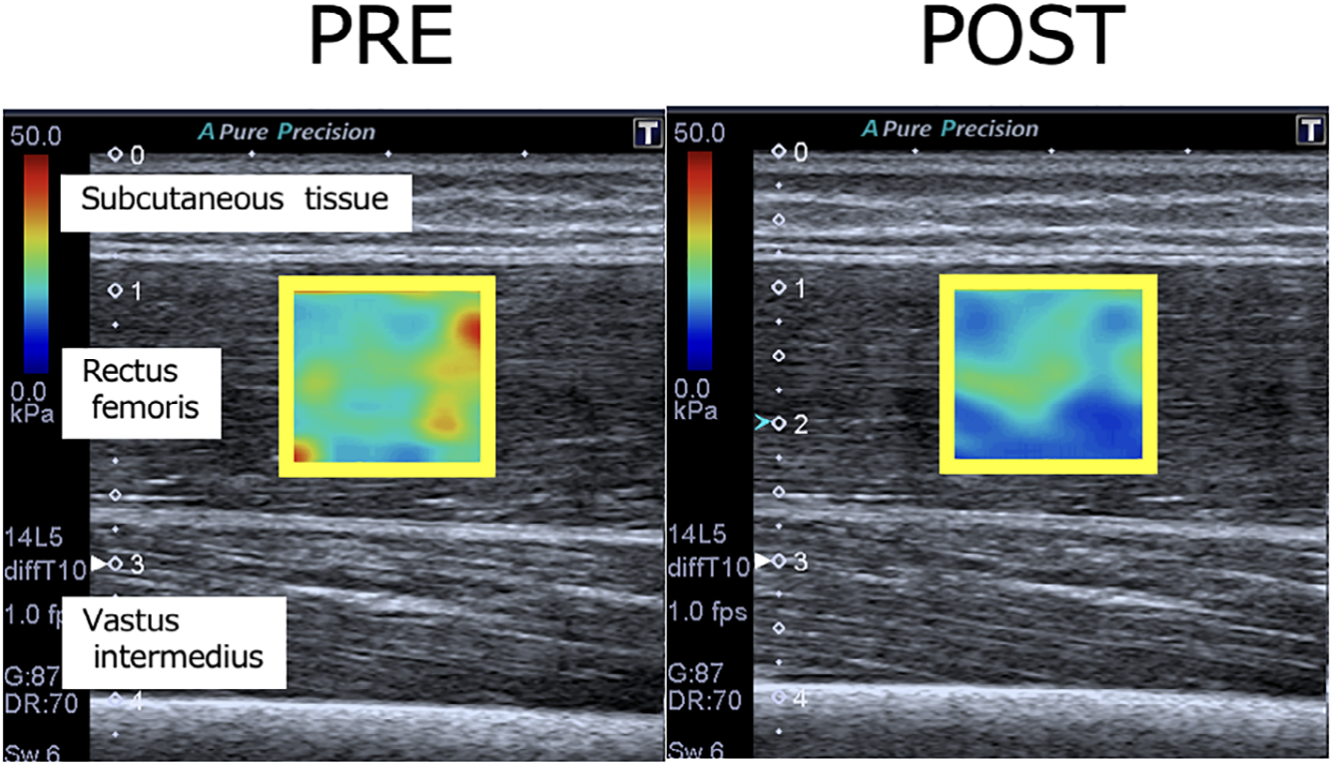
An example of shear‐wave elastography images at PRE and POST from 1 participant (male, 12 years old, height 152 cm, body weight 44.5 kg, shear modulus decreased from 23.5 kPa [PRE] to 18.4 kPa [POST]). The color in the region of interest shown by a yellow square is changed from yellow/green to blue, and the shear modulus decreases after passive movement.

No significant differences were observed between the PRE values of all measurement markers. An interaction was observed between condition and time in the RF shear modulus (*P* < .001) (Figure 4), with significant decreases in the intervention group (*P* < .001) but not in the control group (*P* = .11) (Table 2). In addition, an interaction between condition and time was observed for HBD and subjective pain level (HBD: *P* < .001; subjective pain level: *P* < .001), with decreases in the intervention group (HBD: *P* < .001; subjective pain level: *P* < .001) but not in the control group (HBD: *P* = .19; subjective pain level: *P* = .22) (Table 2).

**TABLE 2:** Changes in Shear Modulus of the RF, HBD, and Subjective Pain Level in Each Condition

	Passive Movement		Control	
	Pre	Post	Pre	Post
RF shear modulus (kPa)	21.0 ± 4.4	17.5 ± 3.2*	20.1 ± 4.4	20.3 ± 4.6
HBD (cm)	12.5 ± 4.7	9.3 ± 3.4*	11.5 ± 4.8	11.5 ± 4.9
Subjective pain level (cm)	4.2 ± 2.4	2.6 ± 2.0*	2.4 ± 2.4	2.3 ± 2.3

*Note*: Values are expressed as mean ± standard deviation.

cm, centimeter; HBD, heel‐buttock distance; kPa, kilopascal; RF, rectus femoris.

* Significantly changed compared with preintervention (*P* < .05).

The intraclass correlation coefficient values for the RF shear modulus measurement variable were 0.99, yielding a standard error of measurement of 0.43 kPa and an MDC_95_ of 1.19 kPa. The mean change in the shear modulus was –3.49 kPa (95% confidence interval: −2.82 to −4.16), with an standardized response mean (SRM) of 1.43 kPa. Depending on the threshold, 83.0% exceeded the MDC_95_ and 86.8% exceeded the SWC (0.2 SD), while 66.0% exceeded the more conservative SWC (0.5 SD). The regression of delta VAS on delta stiffness (*β* = −0.036, *P* = .61) shows that a VAS MCID of 10 to 20 mm corresponds to a stiffness reduction of approximately −0.42 to −0.78 kPa (exploratory MCID).

**FIGURE 5:**
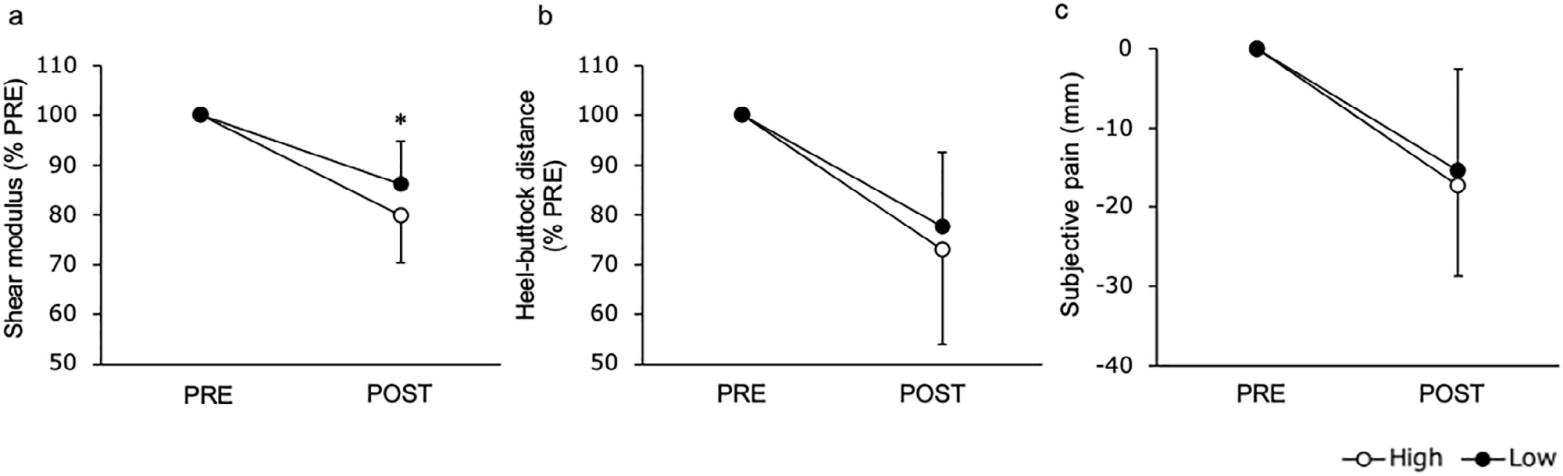
Relative changes in shear modulus, heal‐buttock distance, and subjective pain in the high and low groups after passive movement. Values are expressed as means ± SD. (a) Shear modulus, (b) heal‐buttock distance, and (c) subjective pain, *P < .001. (SD, standard deviation.)

A stratified cluster analysis of the PRE shear modulus of passive movement intervention condition identified 16 and 37 patients in the high‐value and low‐value groups, respectively (high‐value group: 31 males, 12.0 ± 1.9 years, height 154.0 ± 14.9 cm, weight 48.3 ± 12.5 kg; 6 females, 11.7 ± 1.6 years, height 147.8 ± 8.6 cm, weight 37.3 ± 8.8 kg; low‐value group: 16 males, 12.2 ± 2.0 years, height 156.8 ± 10.1 cm, weight 45.1 ± 13.9 kg). The PRE value of the shear modulus in the high‐value group (26.3 ± 2.3 kPa) was significantly higher than in the low‐value group (18.7 ± 2.8 kPa) under passive movement conditions (*P* < .001). The relative change in the shear modulus after passive movement in the high‐value group (−20.1 ± 9.6%) was significantly higher than in the low‐value group (−13.8 ± 8.6%) under passive movement conditions (*P* = .022), but no significant difference was observed between high‐value group and low‐value group for HBD (high‐value group: −27.0 ± 18.9%; low‐value group: −22.3 ± 14.9%, *P* = .33) and subjective pain level (high‐value group: −17.3 ± 11.5%; low‐value group: −15.4 ± 12.9%, *P* = .63) (Figure 5).

**FIGURE 6:**
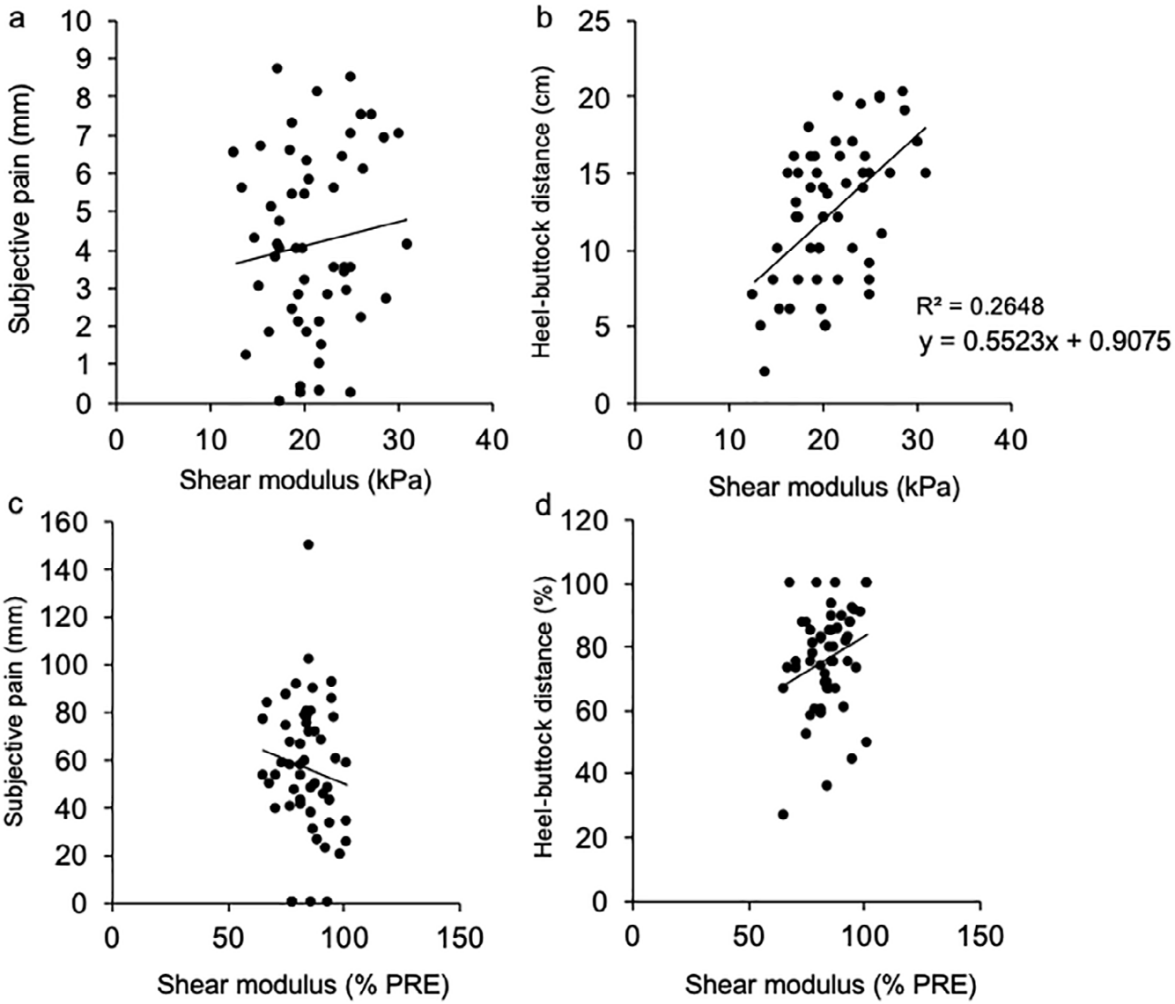
Correlations between shear modulus and subjective pain ((a) raw data; (c) percent change data) and heel‐buttock distance ((b) raw data; (d) percent change data) in the passive movement condition. (kPa, kilopascal.)

Figure 6 presents the results of the correlation analysis between the shear modulus and various other measurements. Positive correlations were observed between the raw shear modulus and raw HBD (*r* = 0.515, *P* < .001); however, no significant correlations were observed between the raw shear modulus and subjective pain level (*r* = 0.004, *P* = .98), the relative change of the shear modulus and relative change of the subjective pain level (*r* = –0.167, *P* = .22), and the relative change of the shear modulus and relative change of the HBD (*r* = 0.250, *P* = .063) (Figure 6).

## DISCUSSION

In this study, 10 minutes of passive hip and knee joint movement was found to reduce RF stiffness in patients with OSD while improving the HBD and subjective pain level of the extended knee joint. Furthermore, passive movement showed the greatest effect in the high‐value shear modulus group compared to the low‐value group.

First, we assessed the muscle condition and verified the validity of the data using the PRE data. The PRE RF shear modulus values were 21.0 ± 4.4 kPa in the intervention group and 20.1 ± 4.4 kPa in the control group, showing no significant difference. When the results of Enomoto et al.,^13^ who used a similar experimental setting and measurement method, were recalculated into the same units (converting shear speed [m/s] to shear modulus [kPa]), the value was approximately 9 kPa; therefore, RF stiffness in this study was approximately twice as high. Furthermore, the intervention in the present study was administered on the day of diagnosis to patients with OSD in various competitive sports; in contrast, Enomoto et al.^13^ included only basketball and volleyball players and up to 4 weeks had passed since the OSD diagnosis. Therefore, the value difference between the studies was likely due to the sports characteristics of the participants and the condition of OSD. Additionally, the results for quadriceps femoris muscle tightness are consistent with earlier reports.^3^, ^28^ These results indicate that the experimental setup used herein can provide useful data for examining OSD. Moreover, in accordance with a previous study^9^ that examined the relationship between growth, muscle stiffness, and OSD, chronological age was not included as a variable in the present analysis. This decision was based on findings indicating that biological maturity, estimated from years and months relative to peak height velocity, was not significantly influenced by age. Furthermore, because anchor methods were not available, we used MDC and SWC in combination to distinguish between “actual change beyond error” and “minimal meaningful change.” Furthermore, we presented an exploratory “estimated MCID” based on an indirect anchor, mapping the previously reported MCID for pain scores to muscle stiffness via regression. This estimate is population‐ and device‐dependent, and future validation using anchor methods with patient‐reported outcomes is necessary.

As an index of RF muscle stiffness in OSD, the shear modulus did not differ between PRE and POST in the control group; however, it significantly decreased after passive movement in the intervention group. This shows that passive movement can acutely improve the stiff RF caused by OSD. In this study, the RF shear modulus improved by 17%, the HBD by 26%, and subjective pain by 38%. The main outcome of this study, i.e., changes in shear modulus, was not inferior to stretching in improving joint flexibility and showed a greater improvement than in previous stretching studies.^29^, ^30^, ^31^ Muscle stiffness affects joint flexibility of the muscle‐tendon unit, and stretching can alter its mechanical and neural properties.^32^, ^33^ Therefore, joint flexibility is expected to improve following a reduction in musculotendinous stiffness. Moreover, the stratified cluster analysis showed that the higher the PRE shear modulus, the greater the improvement of muscle tightness after passive movement. Although the detailed mechanism behind this is difficult to clarify based on the current results, viscoelasticity and/or hysteresis, including slack against the OSD RF after being stretched by passive movement, may be involved. As this is an important clinical trial related to the effectiveness of the treatment, further research is required.

No relationship was found between the PRE RF shear modulus and subjective pain, but a significant relationship with HBD was found. This shows that HBD testing could explain 26% of the RF shear modulus. Muscle stiffness, as measured using SWE, reflects stiffness in the direction of the muscle fibres.^11^ According to Enomoto's report,^13^ knee flexion range of motion is strongly influenced by RF stiffness, and stiff RF muscles affected by OSD become even stiffer than healthy RF muscles. Although these findings may explain the significant association between the RF shear modulus and HBD, the weak correlation suggests that HBD reflects not only the RF but also other surrounding tissues, including the remaining quadriceps muscles, tendons, ligaments, skin, and subcutaneous fat. However, given that SWE‐equipped ultrasound devices are expensive, HBD remains an option for measuring RF stiffness in patients with OSD.

A key role of HBD‐related joint flexibility is its contribution to physical movement; an optimal level of flexibility is required for movements performed during physical exercise and sports activities.^34^ Several studies have examined the importance of RF muscle shortening,^26^, ^35^ which may substantially affect knee biomechanical function through changes in lever arm, peak torque, and the discharge of compressive forces at 30° and 60°.^35^ Nakase et al.^3^ reported that the most important aspect of preventing OSD is improving quadriceps femoris flexibility; however, they observed no association with subjective pain. One reason is that mechanical stimulation from passive movement can inhibit RF muscle activity. Therefore, this may have affected the subjective pain test, which involves contraction of the RF and flexion and extension of the knee. This suggests the importance of objective measurements using SWE or the HBD rather than subjective pain, for which no correlation was found in this study.

Knee flexion to stretch the quadriceps femoris^36^ and stretching the RF with hip extension have long been suggested for children with OSD.^37^ From a patient's perspective, these methods are readily available on the Internet. Considering RF anatomy, this stretching position allows for maximum stretching of the RF. However, because increasing the traction stress on the RF muscle is undesirable, stretching that involves large joint movements, such as full hip extension and maximum knee flexion, may not be recommended depending on the OSD symptoms. Therefore, verifying the acute effects of passive movement on RF stiffness, joint flexibility, and subjective pain of the knee contributes to expanding future rehabilitation methods for OSD patients.

### Limitations

This study has some limitations. Only passive movement was performed on the RF, which had become stiff due to the OSD. Therefore, the magnitude of the effect cannot be compared with that of other techniques. Myoelectrical activity was not recorded to ensure that the muscles remained passive; however, similar to previous studies, participants were verbally instructed to remain relaxed before each knee flexion. The researcher monitored the B‐mode images during each measurement. If any muscle contraction was detected in the B‐mode images, the data were discarded, and another measurement was performed. In addition, due to the small number of females in our study population, we were unable to disaggregate data by sex to determine its impact.

## CONCLUSIONS

Ten minutes of passive hip and knee joint movement reduced RF stiffness in patients with OSD and improved knee joint flexibility and subjective pain. Furthermore, passive movement showed the greatest effect in the high‐value shear modulus group.

## DISCLOSURES

The authors (T.I., N.I., S.Y.) declare the following financial interests/personal relationships which may be considered as potential competing interests: T.I. reports financial support provided by Yurashi International, Japan. N.I. reports financial support provided by Yurashi International, Japan. S.Y. reports financial support provided by Yurashi International, Japan. The other author (G.K.) declares that he has no known competing financial interests or personal relationships that could have appeared to influence the work reported in this article.
